# Supplementary material for: Regulation of the physiology and virulence of Ralstonia solanacearum by the second messenger 2′,3′-cyclic guanosine monophosphate
Source: Nat Commun. 2023 Nov 23;14:7654. doi: 10.1038/s41467-023-43461-2 (PMC10667535; doi:10.1038/s41467-023-43461-2)
Supplement: Supplementary file 1 — Supplementary Information [file 41467_2023_43461_MOESM1_ESM.pdf]

Supplementary information

Regulation of the physiology and virulence of *Ralstonia solanacearum* by the second messenger 2',3'-cyclic guanosine monophosphate

Xia Li[1,9], Wenfang Yin[2,9], Junjie Desmond Lin[3,9], Yong Zhang[4], Quan Guo[1], Gerun Wang[1], Xiayu Chen[1], Binbin Cui[1], Mingfang Wang[1], Min Chen[4], Peng Li[5], Ya-Wen He[6], Wei Qian[7], Haibin Luo[8], Lian-Hui Zhang[2], Xue-Wei Liu[3]*, Shihao Song[8]*, Yinyue Deng[1]*

[1]*School of Pharmaceutical Sciences (Shenzhen), Shenzhen Campus of Sun Yat-sen University, Sun Yat-sen University, Shenzhen, China*

[2]*Integrative Microbiology Research Center, College of Plant Protection, South China Agricultural University, Guangzhou China*

[3]*Division of Chemistry and Biological Chemistry, School of Physical and Mathematical Sciences, Nanyang Technological University, Singapore*

[4]*College of Resources and Environment, Southwest University, Chongqing, China*

[5]*Ministry of Education Key Laboratory for Ecology of Tropical Islands, Key Laboratory of Tropical Animal and Plant Ecology of Hainan Province, College of Life Sciences, Hainan Normal University, Haikou, China.*

[6]*State Key Laboratory of Microbial Metabolism, Joint International Research Laboratory of Metabolic and Developmental Sciences, School of Life Sciences and Biotechnology, Shanghai Jiao Tong University, Shanghai 200240, China*

[7] *State Key Laboratory of Plant Genomics, Institution of Microbiology, Chinese Academy of Sciences, Beijing, China.*

[8] *Key Laboratory of Tropical Biological Resources of Ministry of Education, School of Pharmaceutical Sciences, Hainan University, Haikou, China*

[9]The authors contributed equally: Xia Li, Wenfang Yin, Junjie Desmond Lin.

***Corresponding author:**

Xue-Wei Liu: xuewei@ntu.edu.sg

Shihao Song: songsh@hainanu.edu.cn

Yinyue Deng: dengyle@mail.sysu.edu.cn

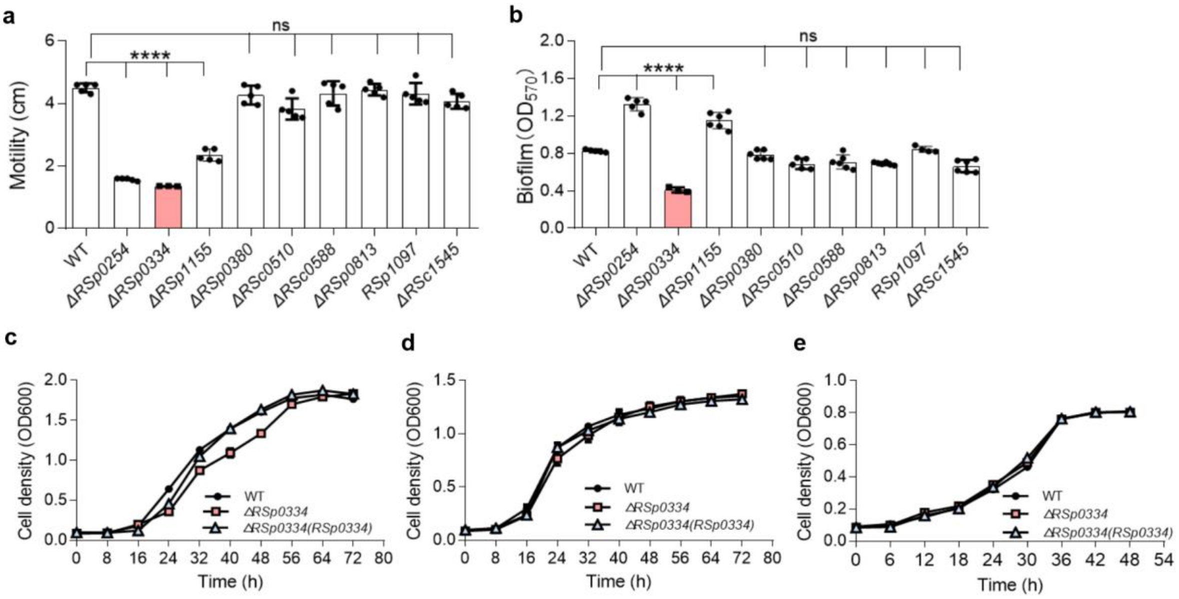

Supplementary Figure 1. Phenotype analysis of potential nucleotide second messenger metabolic enzyme deletion mutants. The mutant strains were constructed by deleting *RSp0254*, *RSp0334*, *RSp1155*, *RSp0380*, *RSc0510, RSc0588*, *RSp0813, RSp1097* and *RSc1545*. **a** The motility of theses mutant strains were measured (n = 5 biological replicates), ****$p<0.0001$. **b** The biofilm formation of these mutant strains were measured (n = 6 biological replicates), ****$p<0.0001$. Cells were inoculated in triplicate at 28°C with low-intensity shaking in a Bioscreen-C automated growth curve analysis system. The experiments were started at an initial OD_{600} of 0.1 in TTC medium **c**, SP medium **d**, and MM medium **e** (n = 3 biological replicates). Data are presented as mean ± SD and are representative of three independent experiments. The statistical comparisons were performed using one-way ANOVA. Source data are provided as a Source Data file.

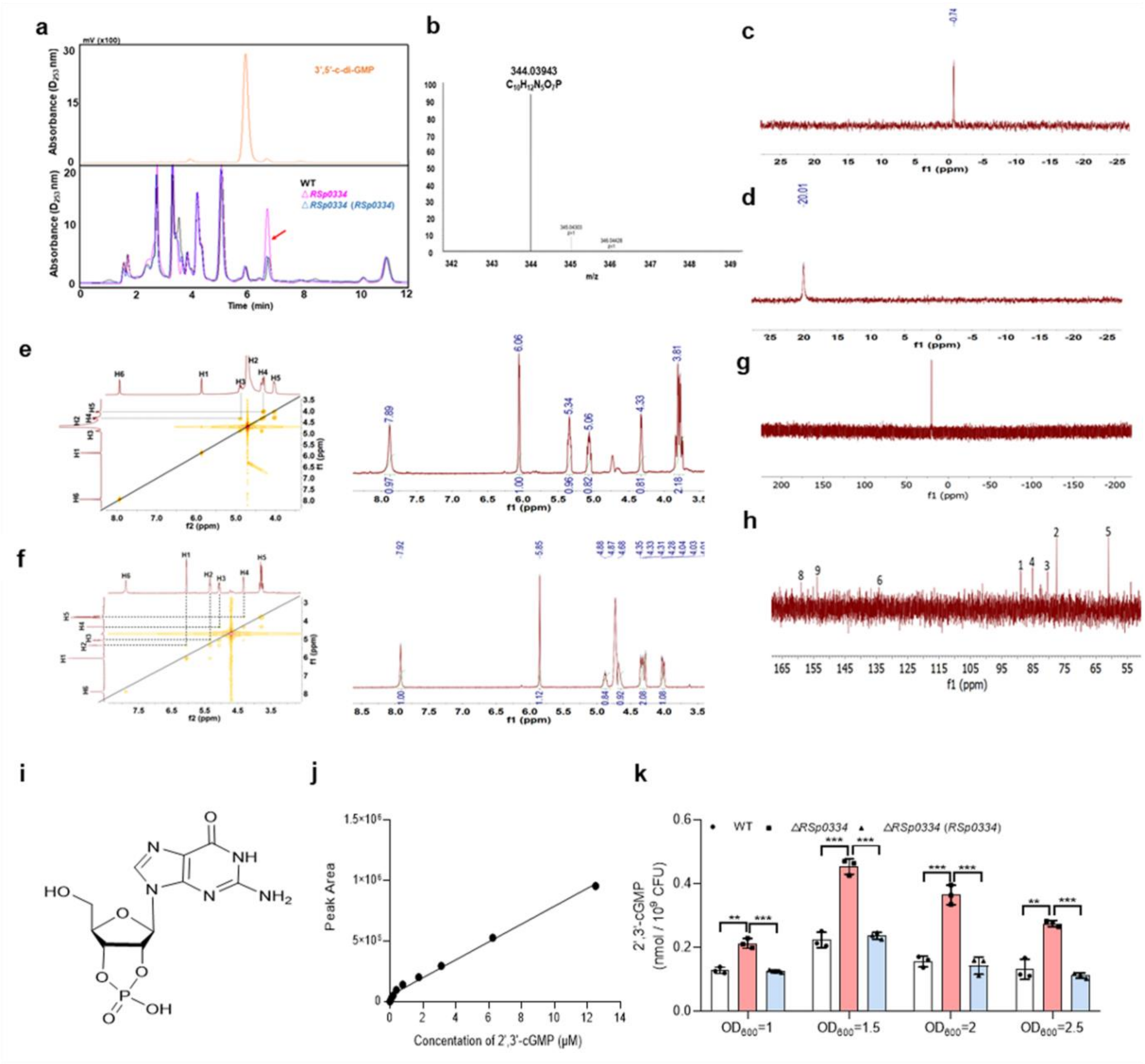

Supplementary Figure 2. Structural characterization of 2',3'-cGMP. a Representative chromatograms of bis-3',5'-c-di-GMP and extracts of *R. solanacearum* wild-type strain (WT) culture (black), Δ*RSp0334* culture (pink), and Δ*RSp0334* (*RSp0334*) culture (blue). **b** ESI-MS spectra (in negative ion mode) of extracts of *R. solanacearum* WT culture. ^{31}P-NMR spectra of bis-3',5'-c-di-GMP **c** and extracts of *R. solanacearum* WT culture **d**. ^{1}H-^{1}H COSY spectrum and ^{1}H-NMR spectrum of extracts of *R. solanacearum* WT culture **e** and bis-3',5'-c-di-GMP **f**. **g** ^{31}P NMR spectra of the standard 2',3'-cGMP. **h** ^{1}H NMR spectra of the standard 2',3'-cGMP. **i** The structure of 2',3'-cGMP. **j** Calibration curves were made by plotting the peak area (Y) versus the concentrations (X, μM) of the standard solutions of 2',3'-cGMP. The regression equation of 2',3'-cGMP was Y = 73883X + 48450, and the linear R^2 was 0.9910. **k** Measurement of 2',3'-cGMP production via LC–MS (*n* = 3 biological replicates), ***p*=0.00127, ****p*=0.00067, ****p*=0.00031, ****p*=0.00014, ****p*=0.00051, ****p*=0.00071, ***p*=0.00162, ****p*=0.00024. Data are presented as mean ± SD and are representative of three independent experiments. The statistical comparisons were performed using one-way ANOVA. In **a**

experiment was performed three times and representative images from one experiment are shown. Source data are provided as a Source Data file.

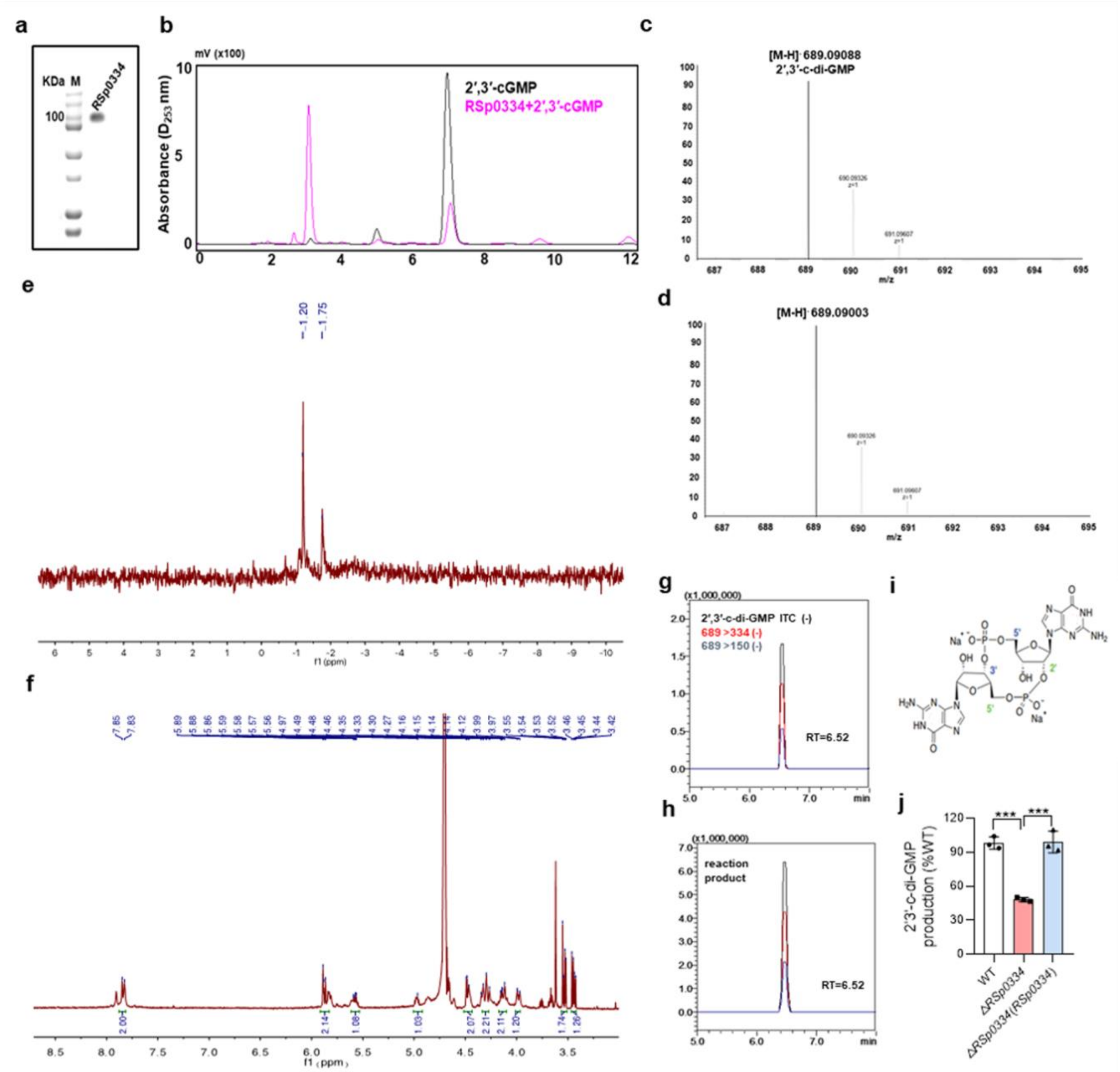

Supplementary Figure 3. *In vitro* enzyme activity assays of RSp0334 on 2',3'-cGMP. a SDS–PAGE of purified RSp0334. **b** RSp0334 has 2',3'-c-di-GMP synthetase activity by using 2',3'-cGMP as the substrate, (2',3'-cGMP: black, RSp0334+2',3'-cGMP: pink). **c** and **d**: comparison of the high-resolution MS spectra (in negative ion mode) of the standard 2',3'-c-di-GMP **c** and the produced 2',3'-c-di-GMP **d**. **e** ^{31}P NMR spectra of the produced 2',3'-c-di-GMP. **f** ^{1}H NMR spectra of the produced 2',3'-c-di-GMP. The triple-quadrupole mass spectra in an MRM model of the standard 2',3'-c-di-GMP **g** and the produced 2',3'-c-di-GMP **h**, the retention time is 6.52 min. **i** The structure of 2',3'-c-di-GMP. **j** Measurement of the intracellular levels of 2',3'-c-di-GMP via LC–MS (*n* = 3 biological replicates). For convenient comparison, 2',3'-c-di-GMP signal production of the *R. solanacearum* GMI1000 wild-type strain was arbitrarily defined as 100% and used to normalize the signal

ratios of other strains, ***p=0.00011, ***p=0.00079. Data are presented as mean ± SD and are representative of three independent experiments. The statistical comparisons were performed using one-way ANOVA. In **a-b** experiment was performed three times and representative images from one experiment are shown. Source data are provided as a Source Data file.

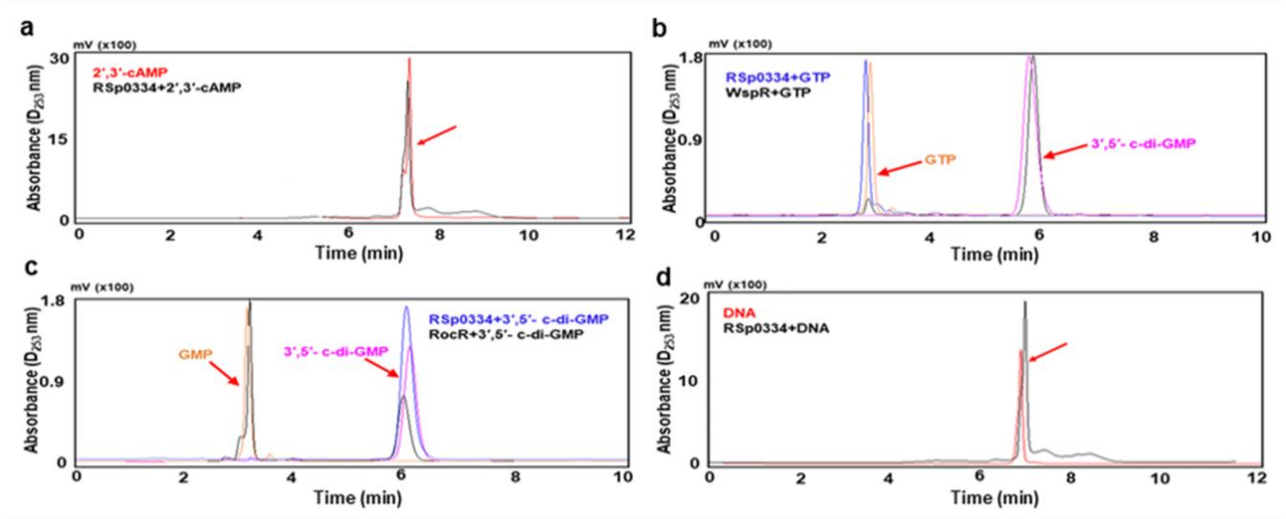

Supplementary Figure 4. *In vitro* enzyme activity assay of RSp0334. a RSp0334 did not exhibit activity on 2',3'-cAMP (2',3'-cAMP: red, RSp0334+2',3'-cAMP: black). **b** RSp0334 did not catalyze the synthesis of bis-3',5'-c-di-GMP after mixing with GTP for 10 min (GTP: orange, bis-3',5'-c-di-GMP: pink, WspR+GTP: black, RSp0334+GTP: blue). **c** RSp0334 did not produce GMP after mixing with bis-3',5'-c-di-GMP for 10 min (GMP: orange, bis-3',5'-c-di-GMP: pink, RocR+bis-3',5'-c-di-GMP: black, RSp0334+3',5'-c-di-GMP: blue). **d** RSp0334 did not produce 2',3'-cGMP when mixed with *R. solanacearum* DNA for 60 min (DNA: red, RSp0334+DNA: black). In **a-d** experiment was performed three times and representative images from one experiment are shown. Source data are provided as a Source Data file.

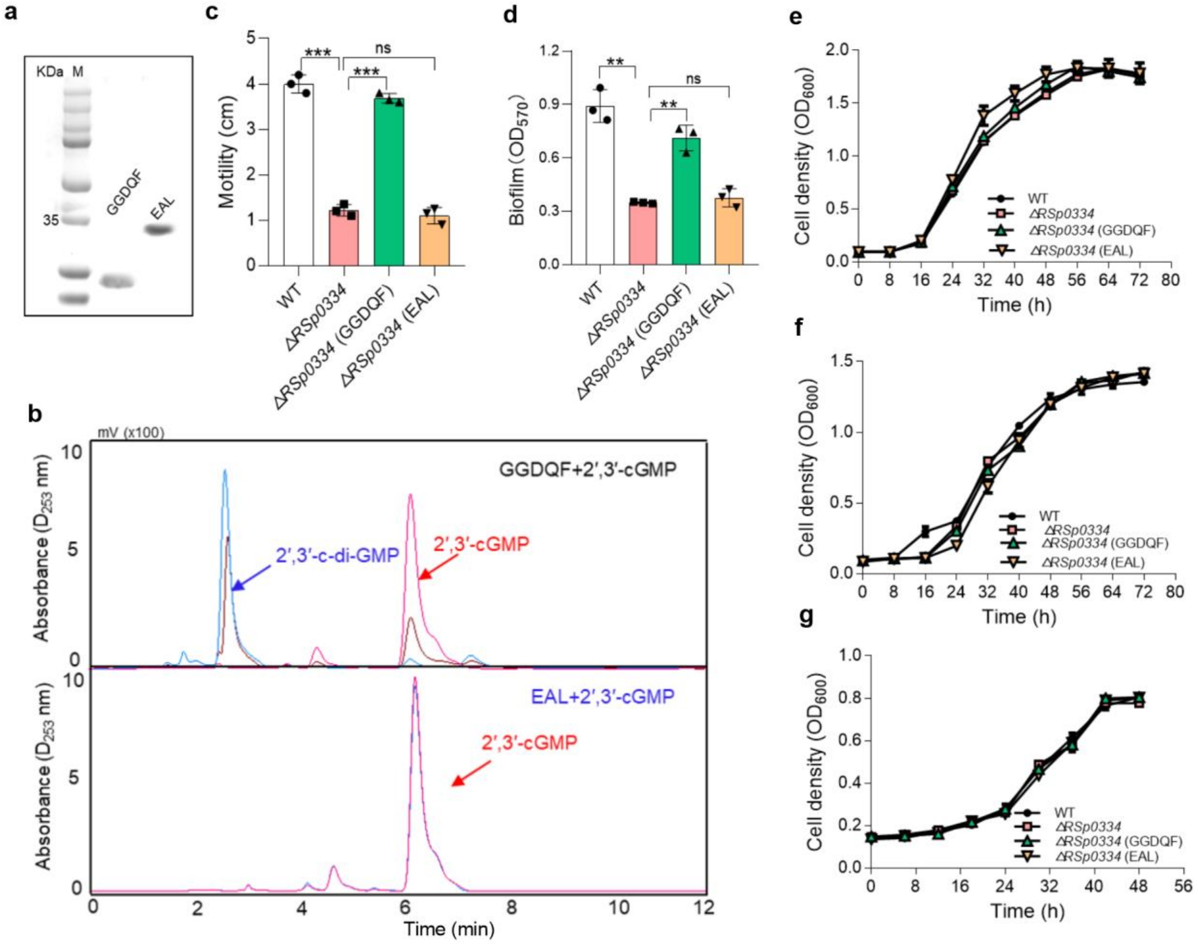

Supplementary Figure 5. Analysis of the function of the RSp0334 domains. a SDS–PAGE of the purified GGDQF domain and EAL domain of RSp0334. **b** The GGDQF domain of RSp0334 efficiently catalyzed the synthesis of 2',3'-c-di-GMP after mixing with 2',3'-cGMP for 10 min (2',3'-cGMP: red, 2',3'-c-di-GMP: blue, GGDQF domain of RSp0334 +2',3'-cGMP: black), whereas the EAL domain of RSp0334 did not (2',3'-cGMP: red, EAL domain of RSp0334 +2',3'-cGMP: blue). **c-d** The wild-type strain, the *RSp0334* mutant strain and the complemented *RSp0334* mutant strains with GGDQF or EAL domains were evaluated for motility and biofilm formation (n = 3 biological replicates). **c** ***p=0.00034, ***p=0.00012, ^{ns}p=0.41; **d** ***p=0.00053, ***p=0.00096, ^{ns}p=0.42. Cells were inoculated in triplicate at 28°C with low-intensity shaking in a Bioscreen-C automated growth curve analysis system. The experiments were started at an initial OD_{600} of 0.1 in TTC medium **e**, SP medium **f**, and MM medium **g** (n = 3 biological replicates). Data are presented as mean ± SD and are representative of three independent experiments. The statistical comparisons were performed using one-way ANOVA. In **a-b** experiment was performed three times and representative images from one experiment are shown. Source data are provided as a Source Data file.

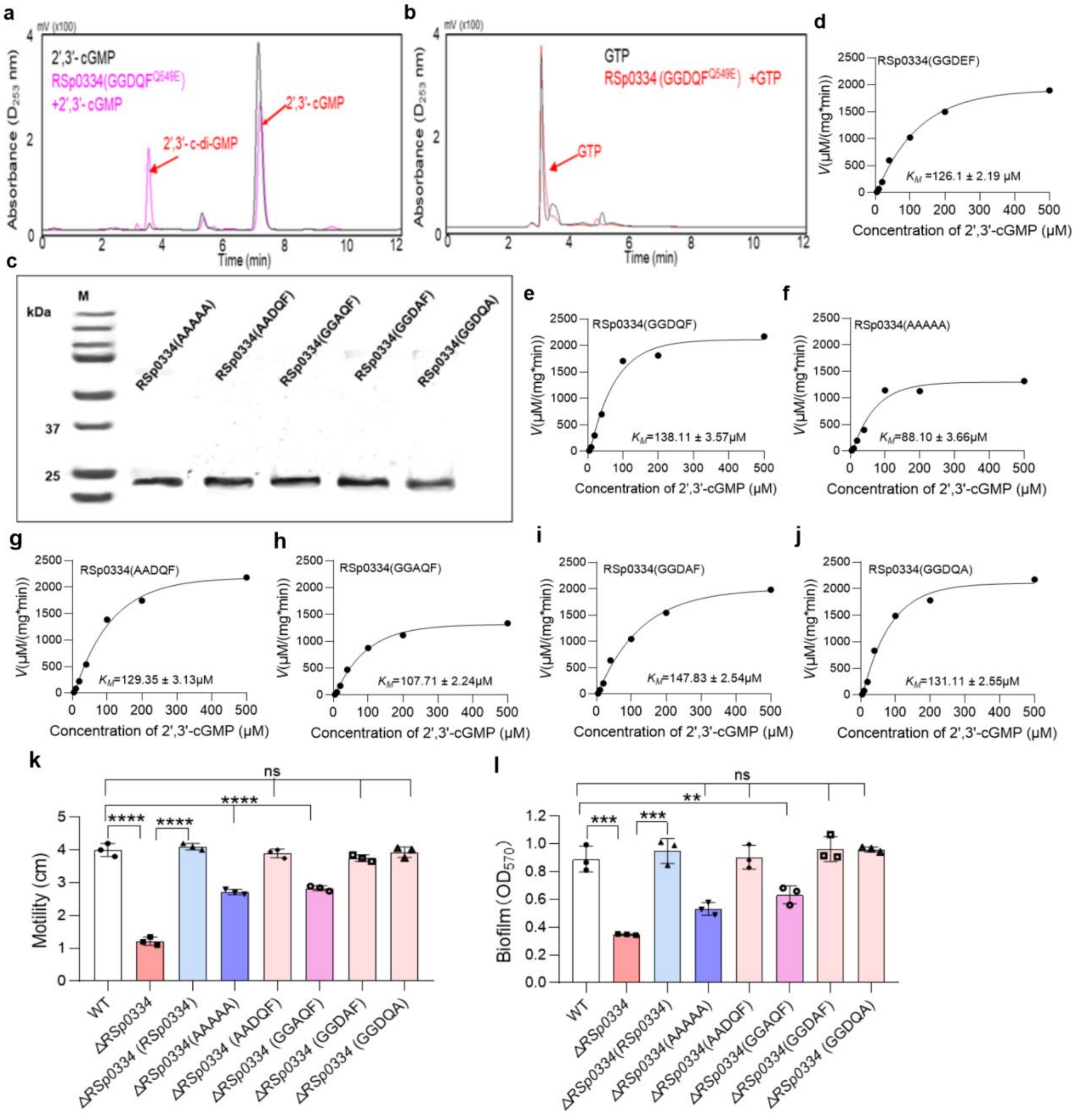

Supplementary Figure 6. Analysis of the enzyme active site of the GGDQF domain of RSp0334 on 2',3'-cGMP. RSp0334 (GGDQF^{Q549E}) retains the ability to convert 2',3'-cGMP to 2',3'-c-di-GMP (2',3'-cGMP: black, RSp0334 (GGDQF^{Q549E}) + 2',3'-cGMP: pink) **a** but does not catalyze the synthesis of 3',5'-c-di-GMP (red) when mixed with GTP (black) for 10 min (GTP: black, RSp0334 (GGDQF^{Q549E}) + GTP: red) **b**. **c** SDS–PAGE of purified RSp0334 (GGDQF) variants. Analysis of the enzyme activity of RSp0334 (GGDQF^{Q549E}) **d**, RSp0334(GGDQF) **e**, RSp0334(AAAAA) **f**, RSp0334(AADQF) **g**, RSp0334(GGAQF) **h**, RSp0334(GGDAF) **i**, and RSp0334(GGDQA) **j** by HPLC. **k-l** *In trans* expression of *RSp0334*(*AAAAA*), *RSp0334*(*AADQF*), *RSp0334*(*GGAQF*), *RSp0334*(*GGDAF*), and *RSp0334*(*GGDQA*) were evaluated for motility and biofilm formation ($n = 3$ biological replicates). **k** ****p<0.00001, ^{ns}p>0.05; **l** ***p=0.00053, ***p=0.00032, **p=0.00223, **p=0.00164, ^{ns}p>0.05. Data are presented as mean ± SD and are representative of three independent experiments. The statistical comparisons were performed using one-way ANOVA. In **a-c** experiment was

performed three times and representative images from one experiment are shown. Source data are provided as a Source Data file.

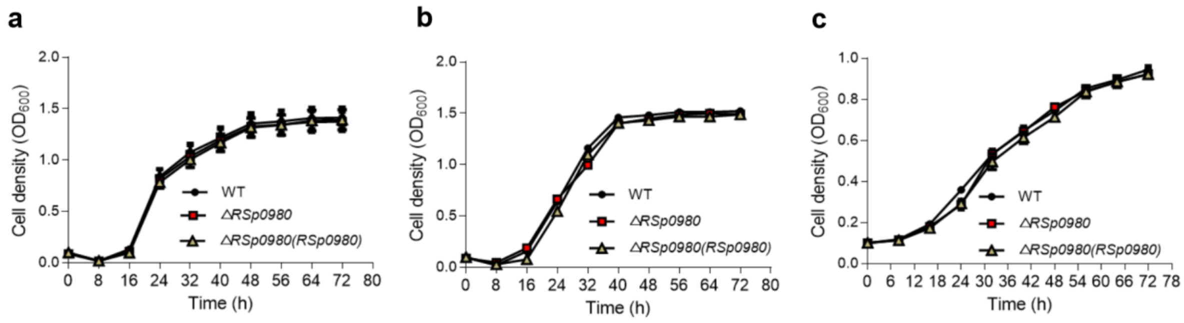

Supplementary Figure 7. Effect of *RSp0980* on the growth of *R. solanacearum*. Cells were inoculated in triplicate at 28°C with low-intensity shaking in a Bioscreen-C automated growth curve analysis system. The experiments were started at an initial OD_{600} of 0.1 in TTC medium **a**, SP medium **b**, and MM medium **c** (*n* = 3 biological replicates). Data are presented as the mean ± standard deviation of three independent experiments. Source data are provided as a Source Data file.

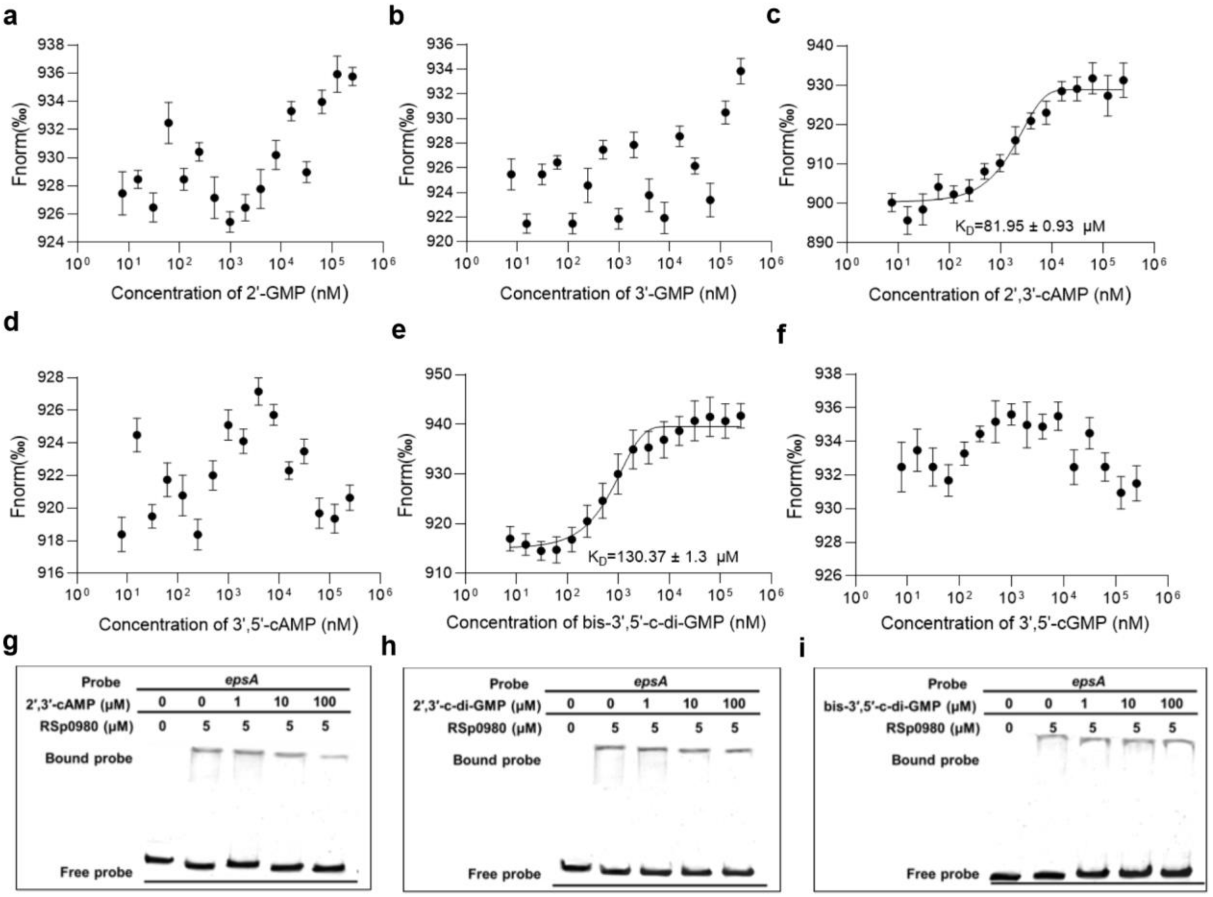

Supplementary Figure 8. Analysis of the binding of RSp0980 to nucleotide molecules. **a-f** MST analysis of the binding of RSp0980 to guanosine-2'-O-monophosphate (2′-GMP), guanosine-3'-O-monophosphate (3′-GMP), 2′,3′-cyclic adenosine monophosphate (2′,3′-cAMP), 3′,5′-cyclic adenosine monophosphate (3′,5′-cAMP), bis-3′,5′-cyclic diguanosine monophosphate (bis-3′,5′-c-di-GMP), and 3′,5′-cyclic guanosine monophosphate (3′,5′-cGMP) (*n* = 4 biological replicates). “Fnorm (‰)” indicates the fluorescence time trace changes in the MST response. The results are presented as the mean ± standard deviation of three independent experiments. EMSA evaluation of the *in vitro* binding of RSp0980 to the promoters of *epsA* with the addition of different amounts of 2′,3'-cAMP **g**, 2 ′,3'-c-di-GMP **h** and bis-3′,5'-c-di-GMP **i**. The protein was incubated with the probe in the presence of different concentrations of 2′,3'-cAMP, 2′,3'-c-di-GMP and bis-3′,5'-c-di-GMP at room temperature for 30 min. In **g-i** experiment was performed three times and representative images from one experiment are shown. Source data are provided as a Source Data file.

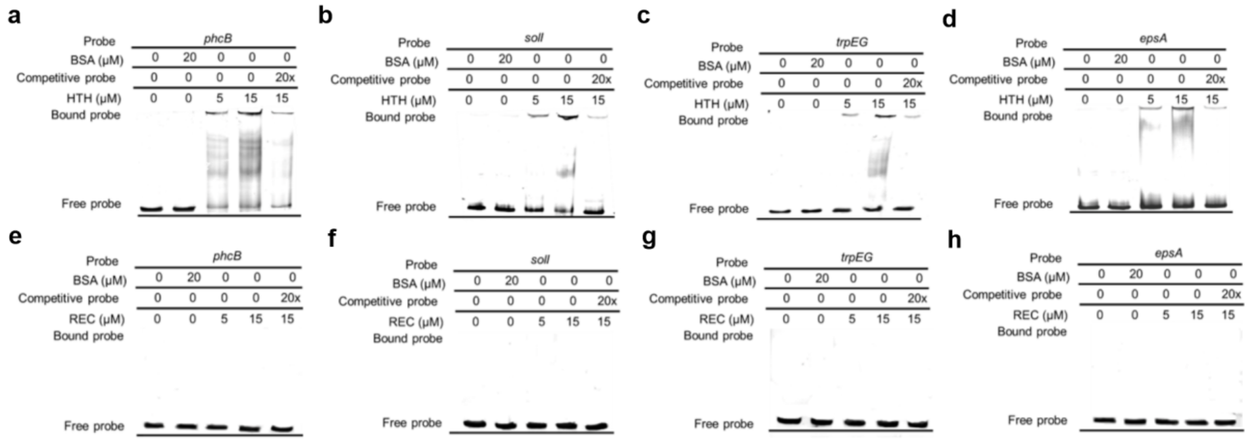

Supplementary Figure 9. Analysis of the DNA binding region in RSp0980 by EMSA. EMSA detection of the *in vitro* binding of the RSp0980-HTH domain to the promoters of *phcB* **a**, *solI* **b**, *trpEG* **c** and *epsA* **d**. EMSA detection of the *in vitro* binding of the RSp0980-REC domain to the promoters of *phcB* **e**, *solI* **f**, *trpEG* **g** and *epsA* **h**. A protein–DNA complex, represented by a band shift, was formed when different concentrations of protein were incubated with the probe at room temperature for 30 min. In **a-h** experiment was performed three times and representative images from one experiment are shown. Source data are provided as a Source Data file.

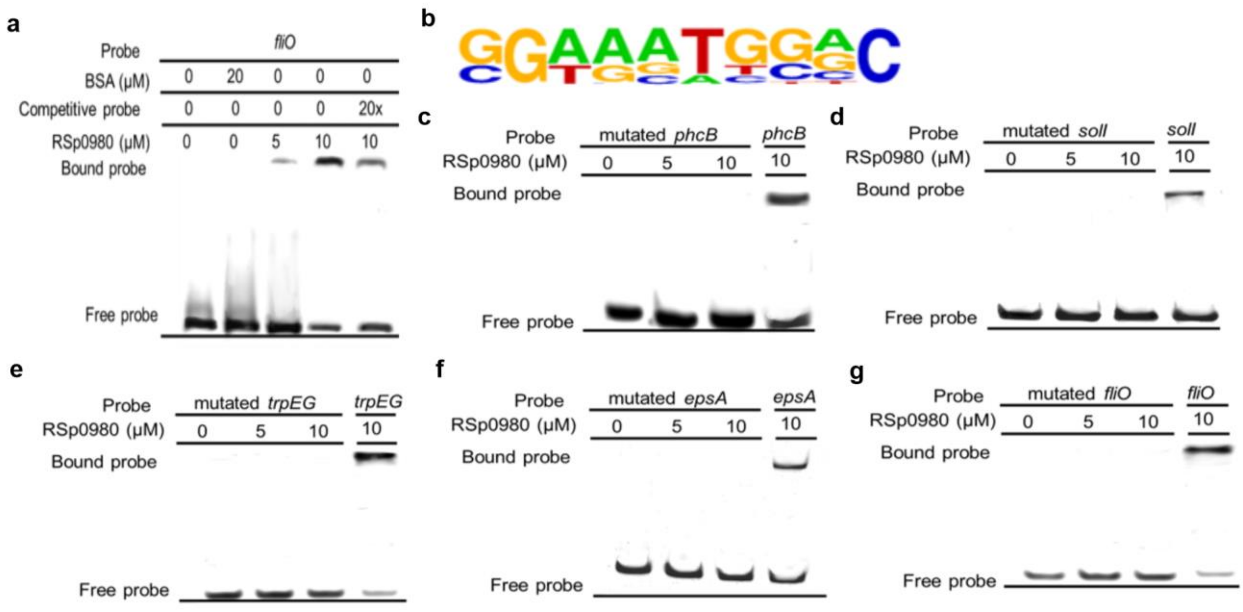

Supplementary Figure 10. EMSA analysis of RSp0980 binding sites in target gene promoters shown in ChIP-seq data. EMSA detection of the *in vitro* binding of RSp0980 to the promoter of *fliO* **a**, the binding sequence of the proposed target gene promoters via ChIP-seq analysis **b**, mutated *phcB* **c**, mutated *solI* **d**, mutated *trpEG* **e** and mutated *epsA* **f**, mutated *fliO* **g**. The potential binding sites 5'-CCTGCCCGAC-3', 5'-GCAAATTCCG-3', 5'-CGAACCCGAC-3', 5'-GGAAGTCGCC-3' and 5'-GGCGCTCGAC-3' were then deleted from the promoter regions of *phcB*, *solI*, *trpEG*, *epsA* and *fliO* respectively. A protein–DNA complex, represented by a band shift, was formed when different concentrations of protein were incubated with the probe at room temperature for 30 min. The experiment was performed three times, representative images from one experiment are shown. Source data are provided as a Source Data file.

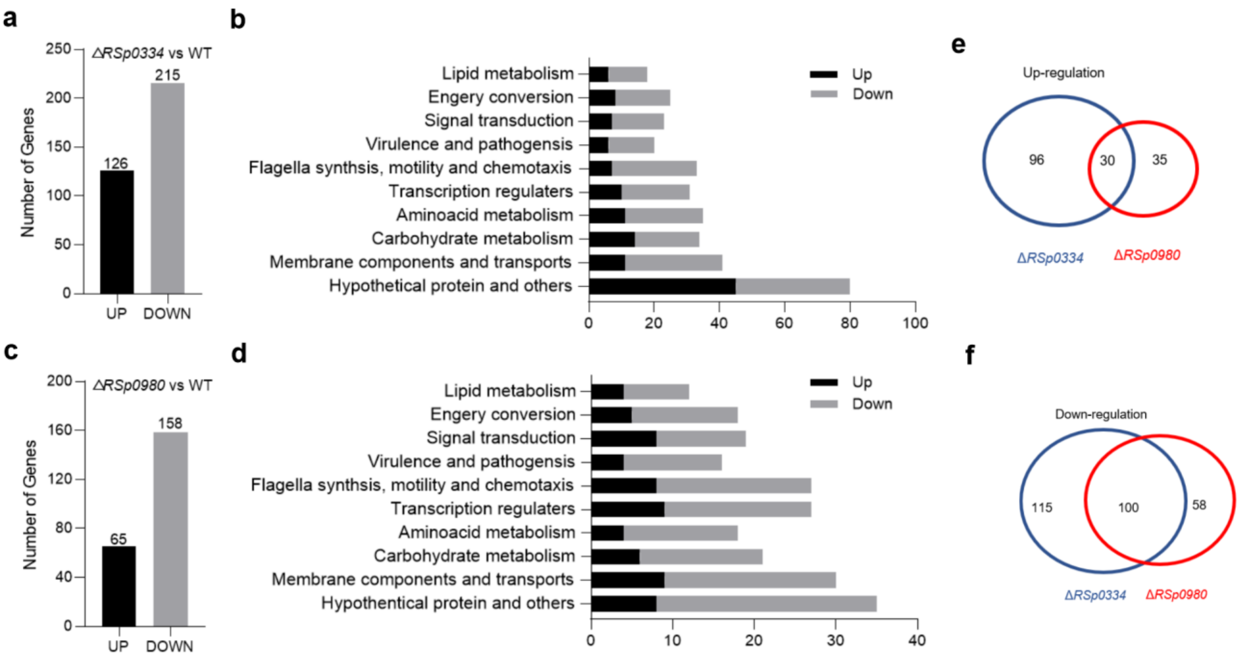

Supplementary Figure 11. Differential gene expression profiles of the *R. solanacearum* wild-type strain, *RSp0334* mutant strain and *RSp0980* mutant strain as measured by RNA-Seq ($\log_2$ fold-change ⩾1.5). a The numbers of genes upregulated (UP) and downregulated (DOWN) in the *RSp0334* mutant strain compared with the wild-type strain. **b** GO term enrichment analysis of differentially expressed genes between the *RSp0334* mutant strain and wild-type strain. **c** The number of genes upregulated (UP) and downregulated (DOWN) in the *RSp0980* mutant strain compared with the wild-type strain. **d** GO term enrichment analysis of differentially expressed genes between the *RSp0980* mutant strain and the wild-type strain. Venn diagrams showing the overlap of genes with **e** upregulated or **f** downregulated expression in different strain backgrounds. Source data are provided as a Source Data file.

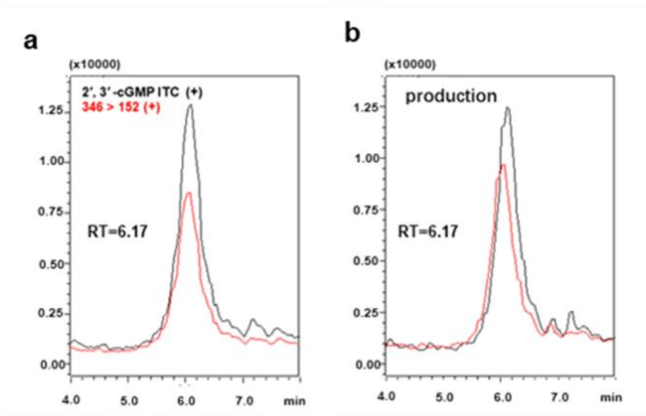

Supplementary Figure 12. *In vitro* enzyme activity assays of RSc2766 on mRNA. The triple-quadrupole mass spectra in an MRM model of the standard 2',3'-cGMP **a** and the produced 2',3'-cGMP **b**, the retention time is 6.17 min. In **a-b** experiment was performed three times and representative images from one experiment are shown. Source data are provided as a Source Data file.

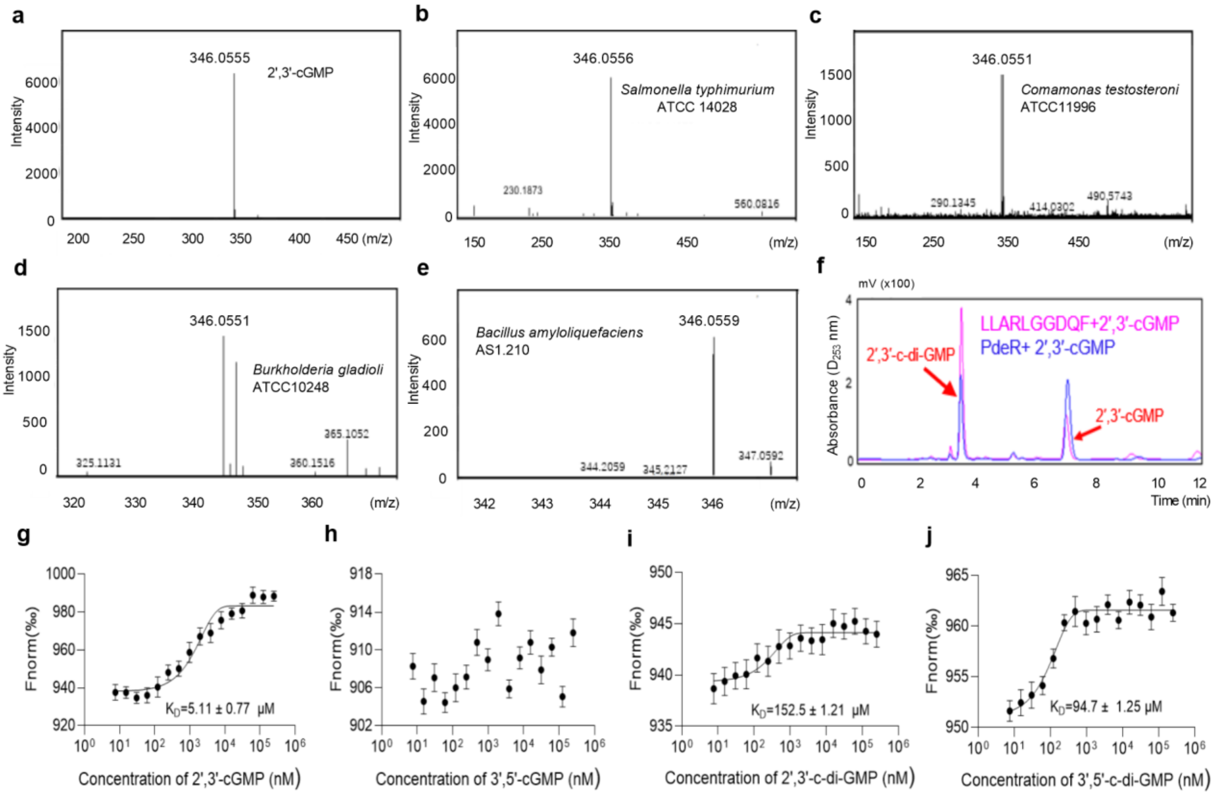

Supplementary Figure 13. Analysis of 2',3'-cGMP in other bacteria. a Chromatograms (in positive ion mode) of the 2',3'-cGMP reference substance sample. **b-e** LC–MS detection of the extracts of *S. typhimurium*, *C. testosteroni*, *B. gladioli*, and *B. amyloliquefaciens*. **f** *S. typhimurium* PdeR (UTL09452.1) converted 2',3'-cGMP to 2',3'-c-di-GMP, similar to the LLARLGGDQF motif of RSp0334 (LLARLGGDQF+2',3'-cGMP: pink, PdeR+2',3'-cGMP: blue). **g-j** MST analysis of the binding of *S. typhimurium* NarL (UTL09391.1) to 2',3'-cGMP, 3',5'-cGMP, 2',3'-c-di-GMP, and bis-3',5'-c-di-GMP (n = 4 biological replicates). "Fnorm (‰)" indicates the fluorescence time trace changes in the MST response. Data are presented as mean ± SD and are representative of three independent experiments. Source data are provided as a Source Data file.

Supplementary Tables

Supplementary Table 1 ^{1}H NMR (400 MHz) and ^{13}C NMR (121 MHz) for compound 2',3'-cGMP in D_2O

**No.**	**δ_C**	**δ_H**	**δ_P**
$-NH_2$			
-NH			
-P(OH)			20.01
-OH			
1	90.3	6.14	
2	81.8	5.44	
3	78.9	5.17	
4	86.5	4.44	
5	62.3	3.93, 3.86 (H_5,$H_{5'}$)	
6	141.5	7.97	
7			
8	160.3		
9	156.4		
10	155.1		

Supplementary Table 2 Analysis of enzymatic kinetic parameters of RSp0334 variants.

Protein	V_{max} (μmol/min·mg)	K_M (μM)
RSp0334(GGDQF)	2.86 ± 0.27	138.11 ± 3.57
RSp0334(AAAAA)	1.64 ± 0.24	88.10 ± 3.66
RSp0334(AADQF)	2.68 ± 0.31	129.35 ± 3.13
RSp0334(GGAQF)	1.65 ± 0.12	107.71 ± 2.24
RSp0334(GGDAF)	2.60 ± 0.18	147.83 ± 2.54
RSp0334(GGDQA)	2.67 ± 0.25	131.11 ± 2.55

Supplementary References

1. Galperin, M.Y. & Chou, S.H. Structural conservation and diversity of PilZ-related domains. *J Bacteriol* **202** (2020).
2. Matsuyama, B.Y. *et al.* Mechanistic insights into c-di-GMP-dependent control of the biofilm regulator FleQ from *Pseudomonas aeruginosa*. *Proc Natl Acad Sci USA* **113**, E209-218 (2016).
3. Whitney, J.C. *et al.* Structure of the cytoplasmic region of PelD, a degenerate diguanylate cyclase receptor that regulates exopolysaccharide production in *Pseudomonas aeruginosa*. *J Biol Chem* **287**, 23582-23593 (2012).
4. Navarro, M.V., De, N., Bae, N., Wang, Q. & Sondermann, H. Structural analysis of the GGDEF-EAL domain-containing c-di-GMP receptor FimX. *Structure* **17**, 1104-1116 (2009).
5. Ma, Q., Yang, Z., Pu, M., Peti, W. & Wood, T.K. Engineering a novel c-di-GMP-binding protein for biofilm dispersal. *Environ Microbiol* **13**, 631-642 (2011).
6. Ozaki, S. *et al.* Activation and polar sequestration of PopA, a c-di-GMP effector protein involved in *Caulobacter crescentus* cell cycle control. *Mol Microbiol* **94**, 580-594 (2014).
7. Krasteva, P.V. *et al. Vibrio cholerae* VpsT regulates matrix production and motility by directly sensing cyclic di-GMP. *Science* **327**, 866-868 (2010).
8. Chakrabortty, T., Roy Chowdhury, S., Ghosh, B. & Sen, U. Crystal structure of VpsR revealed novel dimeric architecture and c-di-GMP binding site: mechanistic implications in oligomerization, ATPase activity and DNA binding. *J Mol Biol* **434**, 167354 (2022).
9. Wang, Y.C. *et al.* Nucleotide binding by the widespread high-affinity cyclic di-GMP receptor MshEN domain. *Nat Commun* **7**, 12481 (2016).
10. Petters, T. *et al.* The orphan histidine protein kinase SgmT is a c-di-GMP receptor and regulates composition of the extracellular matrix together with the orphan DNA binding response regulator DigR in *Myxococcus xanthus. Mol Microbiol* **84**, 147-165 (2012).
11. Yang, F. *et al.* The degenerate EAL-GGDEF domain protein Filp functions as a cyclic di-GMP receptor and specifically interacts with the PilZ-domain protein PXO_02715 to regulate virulence in *Xanthomonas oryzae pv. oryzae*. *Mol Plant Microbe Interact* **27**, 578-589 (2014).
12. An, S.Q. *et al.* Novel cyclic di-GMP effectors of the YajQ protein family control bacterial virulence. *PLoS Pathog* **10**, e1004429 (2014).
13. Ye, P. *et al.* Proline utilization A controls bacterial pathogenicity by sensing its substrate and cofactors. *Commun Biol* **5**, 496 (2022).
14. Shen, F. *et al. Ralstonia solanacearum* promotes pathogenicity by utilizing l-glutamic acid from host plants. *Mol Plant Pathol* **21**, 1099-1110 (2020).
15. Song, S. *et al.* Anthranilic acid from *Ralstonia solanacearum* plays dual roles in intraspecies signalling and inter-kingdom communication. *ISME J* **14**, 2248-2260 (2020).
