# Supplementary material for: Regulation of the physiology and virulence of Ralstonia solanacearum by the second messenger 2′,3′-cyclic guanosine monophosphate
Source: Nat Commun. 2023 Nov 23;14:7654. doi: 10.1038/s41467-023-43461-2 (PMC10667535; doi:10.1038/s41467-023-43461-2)
Supplement: Supplementary file 3 — Description of Additional Supplementary Files [file 41467_2023_43461_MOESM3_ESM.pdf]

Description of Additional Supplementary Data Files

File Name: Supplementary Data 1.

Description: Domain structure, I-site and inhibitory site analysis of bis-3',5'-c-di-GMP metabolic enzymes in *R. solanacearum*. The motifs were predicted by amino acid sequence alignment with RocR or WspR of *Pseudomonas aeruginosa*.

File Name: Supplementary Data 2.

Description: Analysis of the homologs of RpfR in *R. solanacearum* GMI1000.

File Name: Supplementary Data 3.

Description: Homologs of bis-3',5'-c-di-GMP receptor in *R. solanacearum* GMI1000.

File Name: Supplementary Data 4.

Description: Potential target genes regulated by RSp0980 identified via ChIP-seq analysis.

File Name: Supplementary Data 5.

Description: List of genes differentially expressed in the *RSp0334* mutant and *RSp0980* mutant compared to the *R. solanacearum* GMI1000 wild-type strain (Log_2-fold change ≥ 1.5). Significantly differentially expressed genes were determined by Cufflinks after Benjamini-Hochberg correction. The fold-change is the ratio of the mutant FPKM to the wild-type FPKM.

File Name: Supplementary Data 6.

Description: Bacterial strains and plasmids used in this study.

[a] Kan^r, Tet^r, and Amp^r indicate resistance to kanamycin, tetracycline and ampicillin, respectively.

File Name: Supplementary Data 7.

Description: PCR primers used in this study.
